# Irrigation, migration and infestation: a case study of Chagas disease vectors and bed bugs in El Pedregal, Peru

**DOI:** 10.1590/0074-02760240002

**Published:** 2024-09-02

**Authors:** Raquel Gonçalves, Kathryn P Hacker, Carlos Condori, Sherrie Xie, Katty Borrini-Mayori, Lina Mollesaca Riveros, Roger Quispe Apaza, Manuel Ysidro Arratea, Gustavo Nativio, Ricardo Castillo-Neyra, Valerie A Paz-Soldan, Michael Z Levy

**Affiliations:** 1 Universidad Peruana Cayetano Heredia, School of Public Health and Administration, One Health Unit, Zoonotic Disease Research Lab, Lima, Peru; 2 University of Pennsylvania, Department of Biostatistics, Epidemiology and Informatics, Philadelphia, PA, United States of America; 3 University of Michigan, Department of Epidemiology, Ann Arbor, MI, United States of America; 4 Tulane University School of Public Health and Tropical Medicine, Department of Tropical Medicine, New Orleans, LA, United States of America

**Keywords:** migration, Triatoma infestans, bed bugs, disease vectors, Peru

## Abstract

**BACKGROUND:**

The city of El Pedregal grew out of a desert, following an agricultural irrigation project in southern Peru.

**OBJECTIVES:**

To describe infestation patterns by triatomines and bed bugs and their relationship to migration and urbanization.

**METHODS:**

We conducted door-to-door entomological surveys for triatomines and bed bugs. We assessed spatial clustering of infestations and compared the year of construction of infested to un-infested households. To gain a better understanding of the context surrounding triatomine infestations, we conducted in-depth interviews with residents to explore their migration histories, including previous experiences with infestation*.*

**FINDINGS:**

We inspected 5,164 households for *Triatoma infestans* (known locally as the *Chirimacha*); 21 (0.41%) were infested. These were extremely spatially clustered (Ripley’s K p-value < 0.001 at various spatial scales). Infested houses were older than controls (Wilcoxon rank-sum: W = 33; p = 0.02). We conducted bed bug specific inspections in 34 households; 23 of these were infested. These were spatially dispersed across El Pedregal, and no difference was observed in construction age between bed bug infested houses and control houses (W = 6.5, p = 0.07).

**MAIN CONCLUSIONS:**

The establishment of agribusiness companies in a desert area demanded a permanent work force, leading to the emergence of a new city. Migrant farmers, seeking work opportunities or escaping from adverse climatic events, arrived with few resources, and constructed their houses with precarious materials. *T. infestans*, a Chagas disease vector, was introduced to the city and colonized houses, but its dispersal was constrained by presence of vacant houses. We discuss how changes in the socioeconomic and agricultural landscape can increase vulnerability to vector-borne illnesses.

Some great cities of the world have mythical origin stories; the origins of others are more mundane. In many cases the impetus for a *de novo* city is political - national capitals like Washington DC^1^ and Brasilia^2^ were placed in central locations, and the blank slates allowed for national architectural expression.^1^ In many other cases, cities grow *de novo* in response to the exploitation or cultivation of new economic resources, such as rubber (Manaus, Belem, Iquitos),^3^ gold (San Francisco),^4^ and oil (Houston),^5^ among many others. The rapid emergence of entirely new cities allows for the close study of the processes of arrival and spread of urban pests, including disease vectors.

The foundation of new cities increases human migration, mainly as a workforce, and can act as a persistent social determinant in the processes of introduction and emergence of vector-borne diseases.^6^ ^,^ ^7^ Further landscape changes, such as expansion of agricultural frontiers, and changes in land use, provide broad opportunities for new contacts among hosts, vectors, and pathogens.^8^ Socioeconomic factors and the poorly-planned occupation of spaces associated with poverty, are structural processes that can promote conditions for infestation by triatomine bugs, the vectors of the parasite *Trypanosoma cruzi*, causative agent of Chagas disease. Poor housing conditions, such as poor quality construction materials, cracks on the walls, unplastered surfaces, and presence of animals are factors associated with increased risk of triatomine infestation.^9^ ^,^ ^10^ Historically, the application of indoor residual spraying (IRS) has been the main strategy to control triatomine domestic infestation. The INCOSUR-Chagas (Southern Cone Initiative for the Elimination of *Triatoma infestans* and the Interruption of Transfusional Transmission of American Trypanosomiasis) successfully controlled *T. infestans* in many areas across South America.^11^ However, different factors affect IRS efficacy, including its suboptimal implementation,^12^ reduced susceptibility/resistance of triatomine populations to insecticides,^13^ and the use of certain construction materials.^14^

In southern Peru, a new city, El Pedregal, was created *de novo* following an irrigation project that created numerous job opportunities and attracted migrants from local towns as well as more distant regions. We studied the early stages of emergence of one of the main vectors of Chagas disease, *T. infestans*, in El Pedregal as well as bed bugs, *Cimex* sp., which are competent vectors of *T. cruzi*.^15^ ^,^ ^16^ ^,^ ^17^ We performed door-to-door entomological surveys throughout El Pedregal, and, in concert with targeted insecticide application, conducted in-depth interviews with affected residents and their neighbors to examine the economic and social forces that led to the rapid growth of the city in a barren desert.

## MATERIALS AND METHODS

*Study site* - The study was conducted in Villa El Pedregal (-16.32699, -72.19480), Distrito de Majes, Provincia de Caylloma, Departamento de Arequipa, Perú [Supplementary data (Figure)]. Located in the Subtropical Coastal desert^18^ the city sits at an altitude of around 500 m, and boasts a year-round temperate climate (mean annual temperature of 19ºC), but with very little precipitation (150 mm annual mean).^19^ In 2017, the Peruvian census for Majes district reported 60,108 inhabitants.

In the 70’s, the Peruvian Government initiated an irrigation project, ‘*Proyecto Especial Majes-Siguas*’, with the goal of providing a water source for the irrigation of 60,000 hectares destined to agroindustry in the ‘Pampas de Majes’. The first phase of the project consisted of the construction of the Condoroma dam, and channelling (and in some cases tunnelling) water from the Colca River to the Siguas River, and from there to Pampas de Majes. By the 1980s the project had supported the establishment of agroindustry and processing plants in El Pedregal. These in turn attracted subsistence farmers from different parts of Peru, who came to the region seeking work opportunities.^20^

*Entomological surveys* - In collaboration with the Ministry of Health of Arequipa we conducted at-the-door interviews and entomological inspections in El Pedregal between October, 2019 and April, 2022, with numerous disruptions due to the coronavirus disease 2019 (Covid-19) pandemic. These surveys were meant to assess the distribution of triatomine vectors for the purposes of planning IRS to eliminate them. We asked several questions at the door regarding individuals’ experience with triatomines and bed bugs. We then requested permission to perform a full inspection of the house, both indoors and outdoors, for triatomines. Entomological surveys were carried out by trained collectors spending about 30 min in each household (1 person/30 min), including peridomestic areas, depending on the size of the household and number of peridomestic sites. Insects were transported to the Zoonotic Disease Research Laboratory in the city of Arequipa (a 1.5 h drive) at the end of each day where they were counted. Trained laboratory personnel determined the stage of each nymph and sex of adult bugs. Gut contents of all insects, both triatomines and bed bugs, were examined for the presence of *T. cruzi* following our standard protocols.^21^ We separately offered to inspect beds and furniture for bed bugs to those individuals who reported bites or suspected an infestation, following our protocols used in the United States.^22^ Prior to the entomological surveys, we created a georeferenced map of all households and other structures and integrated entomological data into this map in real time using Vectorpoint ― an app created by our research team for use by house inspectors to guide entomological searches.^23^ We informed the Ministry of Health of all households in which we detected triatomines, and, in coordination with them, scheduled these households and their immediate neighbors for insecticide treatment per Ministry of Health guidelines.

*IRS* - IRS was offered to all households where triatomine infestation was confirmed. In the blocks in which infestation was confirmed in more than one household, IRS was offered to all houses within that block, and to households in the neighboring blocks that faced the treated block.^24^ IRS was conducted twice per household at a target 6-month interval, though the pandemic led to a longer delay between applications. Householders were asked to empty their houses and animal enclosures of food and water reservoirs and to move any objects away from walls. IRS was conducted treating all the wall surfaces (indoors and outdoors) of the house and all peridomestic areas, including animal enclosures*.* The first phase (IRS-1) was conducted between November 20th, 2020 and March 17th, 2021; 135 households were treated. The second phase (IRS-2) was conducted between October 5th, 2021 and February 13th, 2022, and encompassed 118 households. All participating households were treated with Lambda-cyhalothrin SC 10% (Arpon^®^ 10 SC) in a dilution of 60 mL of insecticide: 8 Litres of water, according to the manufacture’s indications. The insecticide was provided by the Ministry of Health. Vector control technicians applied the insecticide using a Hudson^®^ sprayer tank (H.D. Hudson Manufacturing Company, Chicago, IL), with 8.5L capacity (Pressure 60 P.S.I.; 414 kPA). There were no official records of insecticide spray campaigns in El Pedregal prior to our study.

**Fig. 1:**
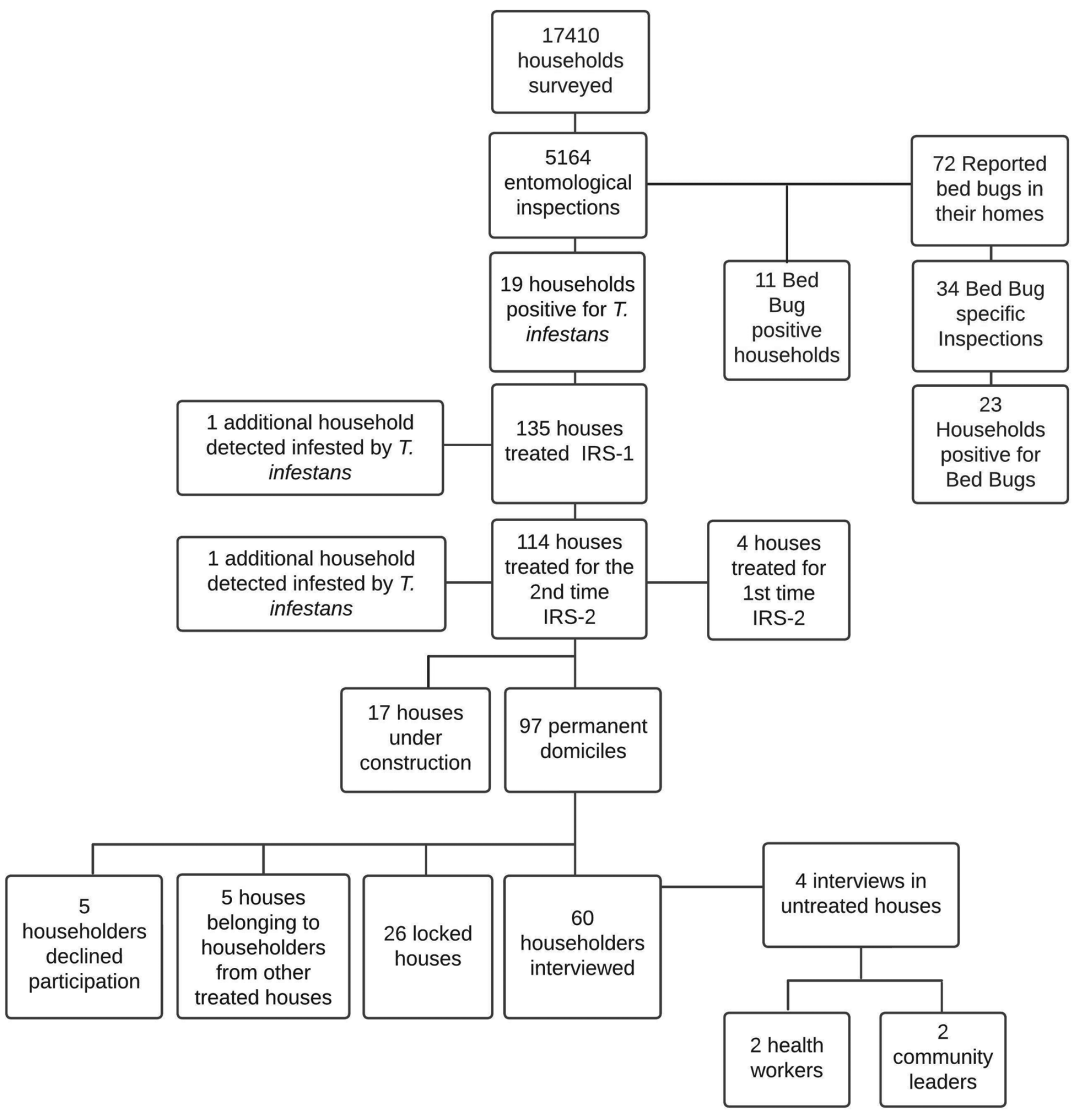
flow chart of entomological activities and in-depth interviews conducted in El Pedregal.

*Semi-structured interviews* - We visited 97 houses (of 118) treated during the 2nd IRS application. Houses were visited up to three times, in different work hours and during weekends. A total of 65 householders were contacted, and individuals 18 years or older were invited to participate in semi-structured interviews. Sixty individuals agreed to participate, while five declined participation. Additionally, two community health workers and two community leaders were invited to participate to provide additional context surrounding vector infestation and control in the region (Fig. 1).

A semi-structured interview guide was developed to ensure that all interviews covered similar topics and was organized in three main areas of interest: 1) migration history; 2) resident perceptions regarding the city’s growth; and 3) experiences and knowledge related to dangerous insects, specifically focusing on triatomines and bed bugs. All interviews were recorded; one of us (RG) listened to each at least twice to add detail to handwritten field notes. A matrix summarizing themes that emerged inductively associated to the three main areas of interest was created manually; this matrix was used to generate the summary of themes and quantify how often these topics emerged. For additional detail and sample quotes, 35 audios were transcribed (n = 25 randomly selected and an additional n = 10 selected by the authors due to their richness), uploaded to Dedoose,^25^ and coded. Coding was both deductive (*i.e.*, based on the main themes explored) and inductive (based on responses that emerged from interviews).^26^ Themes and related quotes are summarized and presented in the results section.

*Assessment of household development via satellite images* - A sample of 142 uninfested houses (2.75% of total surveyed houses n = 5,164) was randomly selected in order to compare the median year of construction with houses infested by *T. infestans*, and with houses infested by *Cimex* sp. The estimated statistical power to detect difference between medians was 80% (alpha = 0.05) by application of non-parametric Wilcoxon rank-sum test. The set of houses was assessed in Google Earth images, obtained for all the years available (2004, 2010, 2012, 2017, 2018, and 2020). A matrix was generated, registering for each house the year in which images indicated first evidence of development, defined as the presence of a building covering any portion of the sample city lot. R Code is available at: https://github.com/chirimacha/Goncalves_et_al_MIOC_2024.

*Spatial analysis* - We assessed spatial clustering of triatomine and bed bug infestations using Ripley’s K function, which compares a given point distribution to complete spatial randomness to determine if points are clustered, dispersed, or occur at random.^27^ Separate K functions were used to examine point patterns of cases (inspected houses that were positive for the infestation of interest) and controls (inspected houses that were negative for the infestation of interest) for both triatomines and bed bugs for a distance of 0 to 1000 m. For each infestation type, we calculated the difference between K functions (KD) for cases and controls to assess for clustering of cases while accounting for underlying spatial heterogeneity in the locations of inspected houses; positive KD values indicate significant clustering of cases. Monte Carlo random labeling simulations (n = 999) were performed to determine 95% confidence intervals to assess statistical significance for alpha level = 0.05. Spatial analyses were conducted in R using the spatstat and smacpod libraries.^28^ ^,^ ^29^ For both remote sensing and spatial analysis, only 33 houses infested by *Cimex* sp. were considered, as geographic coordinates of one house were missing.

*Ethical approval* - The study was approved by the ethical review board of the Universidad Peruana Cayetano Heredia (protocol #103096), the University of Pennsylvania (protocol #833122), and the Tulane School of Public Health and Tropical Medicine (#2019-909). Individuals who agreed to participate in the semi-structured interviews signed a consent form, authorizing recording of the interviews. Entomological surveys and insecticide application conducted as part of the Ministry of Health’s control campaign were deemed not human subjects research and were conducted following verbal consent in accord with the Ministry of Health’s usual protocols.

## RESULTS

*Entomological surveys* - We conducted visits to 17,410 households to survey for triatomine bugs. We were able to interview household members at the door of 12,129 houses (69.7%) and enter and inspect 5,164 (29.66%). We confirmed infestation by *T. infestans* in 19. All households infested by *T. infestans*, as well as their immediate neighbors, were requested to allow IRS treatment. During these treatments we detected triatomines in an additional two households, bringing the total number of infested households to 21 (0.41% of those inspected).

During the at-the-door interviews we asked household members about potential infestations with bed bugs. We presented Petri dishes with bed bug and triatomines specimens at different development phases. We found that 619 householders recognized bed bugs, 72 reported that they had seen a bed bug in their house, and 53 reported that they had been bitten by bed bugs in the last year. Of the 53 who reported recent exposure to bed bugs 34 requested bed bug specific inspections (including beds, furniture, and other likely harbourages). We detected bed bugs in 23 of these houses.

Infested triatomine households were extremely spatially clustered; the 21 total infestations were located on only six city blocks. Four of these blocks were themselves clustered together in two neighborhoods, and one household was not obviously associated with either focus of infestation. By contrast households infested with bed bugs were more widely dispersed, though still clustered in some areas.

A total of 258 triatomines were collected in the 21 positive households [median: 5 per household, interquartile range (IQR): 2, 14.5]. Eleven households had insects solely in peri-domestic spaces; five had insects solely inside in domestic spaces, and five had both. Nymphs were captured in 71% of infested houses indicating colonization by the vector. We microscopically examined a total of 215 of the 258 (83.3%) specimens for *T. cruzi* infection (the remaining insects were too damaged or too dry to examine) and all were negative; although sensitivity of microscopy is not perfect.^30^ ^,^ ^31^

Semi-structured interviews

*Socio-demographic and migration patterns* - Table presents participants demographic information, work at arrival in El Pedregal, and migration history. The majority of migrants (53.1%) reported repeated migration movements, living in at least one city different from their origin place before their move to El Pedregal.

**TABLE:** Participants demographic, migration history and work at arrival in El Pedregal

Participants	Median age (IQR)	
Female (n = 44)	39.5 (30.2; 48)	
Male (n = 20)	40 (27; 58)	
Place of origin (Department level)	n (%)	
Apurimac	1 (1.56)	
Arequipa	25 (39.06)	
Arequipa (born in El Pedregal)	5 (7.81)	
Ayacucho	1 (1.56)	
Cusco	12 (18.75)	
Moquegua	1 (1.56)	
Huánuco	1 (1.56)	
Puno	18 (28.13)	
Education level	Female n (%)	Male n (%)
Uncompleted primary	4 (9.1)	2 (10)
Completed primary	3 (6.8)	2 (10)
Uncompleted secondary	3 (6.8)	3 (15)
Completed secondary	11 (25)	5 (25)
Institute/technical education	3 (6.8)	1 (5)
Graduated	4 (9.1)	3 (15)
Missing information	16 (36.4)	4 (20)
Work at arrival in El Pedregal	Female n (%)	Male n (%)
Agriculture	12 (27.3)	8 (40)
Agriculture and commerce	3 (6.8)	0
Agribusiness companies	4 (9.1)	3 (15)
Commerce and services	8 (18.2)	3 (15)
Housewives	8 (18.2)	0
Arrived as child	6 (13.6)	3 (15)
Born in El Pedregal	3 (6.8)	2 (10)
Missing information	0	1 (5)
Migration history		n (%)
Born in El Pedregal and has never left		5 (7.8)
Migrated from their place of origin straight to El Pedregal		21 (32.8)
Migrated through more than 1 local apart from their place of origin before moving to El Pedregal		34 (53.1)
Migrated from their place of origin to El Pedregal, left, and returned to El Pedregal (back-forth)		4 (6.3)

IQR: interquartile range.

*Work at arrival: the agribusiness companies and changes in agricultural practices* - Upon arrival to El Pedregal, about half of the migrants interviewed reported working in agriculture (Table), either in “parcelas” (*i.e.*, a portion of agriculture terrain of 5 hectares that are part of a bigger farming unit) or in the crop companies.

Initially, in the 1980’s, agricultural activities were focused around raising cattle for milk production. The first settlers received credits and imported animals to support this production. Participants also recounted how one of the first agribusiness companies established in El Pedregal controlled the milk price and, together with the high prices of supplies needed for this activity, made it unsustainable for small farmers to compete. Participants recalled having to sell their cattle and dedicate themselves to other agricultural activities.

“…*Al ganado lechero, los colonos al ganado lechero, muy poco al cultivo de plantas. Eran raros los que sembraban zanahorias o cebollas. No se conocía otro cultivo… La mayor parte de gente trabajaba con ganado lechero porque hubo un crédito con el cual a todos los colonos les llegaron vacas, vacas importadas.*”

*“…With dairy cows, the colonists with dairy cows, there was very little agriculture. It was a rare few that grew carrots or onions. There was no other farming… The majority of people worked with dairy cows because there was a credit system that facilitated the colonists to obtain cows, imported cows.”*

“*La gente se dedicaba a la lechería porque esta es una zona lechera, ganadera, a la venta de leche, lo que es engorde de ganado, era la principal fuente de ingreso acá en Irrigación Majes. Lamentablemente el precio que paga la empresa... que tiene el monopolio, claro [ellos] siempre ha estado por acá, y los elevados costos de los insumos se está perdiendo. Muchos se están deshaciendo del ganado, ya no es sostenible mantener ese negocio.*”

*“The people dedicated themselves to dairy because this was a dairy zone; cattle, to the sale of milk, the fattening of cattle, was the principal source of income here in the Majes irrigation. Unfortunately, with the price the company… that had the monopoly, of course [they] always had been here, and the elevated cost of supplies, it was a losing battle. Many gave up on cattle, it’s no longer sustainable to keep up that business.”*

“…*Yo tenía ganado y pensé ¿Qué hago con mi ganado? Porque no te da… La mayoría de amigos que tenían ganados se están deshaciendo de las vacas.*”

“...*I had cattle and I thought ‘What do I do with my cattle? Because they don’t bring in money... Most of my friends that had cattle gave up on the cows.”*

*Reasons for migration to El Pedregal* - Migrants perceived ― and moved to - El Pedregal as a place with opportunities to improve their lives; with more permanent opportunities to work, to build their homes, and to provide education for their children. Some migrated to El Pedregal to reunite with a family member already established there; others migrated from locations with seasonal work activities, such as those from Puno who mentioned that agriculture and cattle raising was seasonal, compared to opportunities in El Pedregal where the cultivation of different plant species provided year-round employment.

*“…Porque en mi tierra (Puno) no hay mucho trabajo. Acá en Pedregal hay trabajito, de todos lados... Allá solamente me dedicaba con mis ganados. Miraba mi ganado porque no había mucho trabajo. El ganado se vende anual, no es así mensual... Entonces también tengo unos cinco hijos y me he venido para acá para sustentar a mis hijos, para hacerlos estudiar.”*

*“...Because where I am from (Puno), there is not much work. Here in Pedregal there are jobs, of all kinds… There [in Puno] I only dedicated myself to my livestock. I would watch my livestock because I did not have much work. Livestock is sold annually, not monthly… I also have five children and I have come here to be able to support my children, to have them study.”*

The demand for goods increased as the population increased, boosting the local economy, and generating additional work opportunities in commerce, construction, and various service industries. The stability of these permanent year-round labor opportunities combined with the possibility to participate in “loteamiento” (procurement of land lots) in El Pedregal, were reported as reasons for migration.

Climatic conditions were also mentioned as a reason for migration. In Puno, for example, climate events such as floods affect crops, lives, and food security in recent years;^32^ the climate in El Pedregal is considered appropriate year-round for different agricultural activities.

*“…Allá (Puno) sufríamos inundaciones... todo se ha llevado la cosecha por eso nos hemos venido, buscando trabajo y hemos llegado… Ahora se están inundando por ahi…”*

*“…Over there [Puno] we suffered from flooding… floods have taken all the crops, that is why we have come, we arrived looking for work… Now everything is being flooded over there…”*

“*Primeramente, esta es una irrigación, o sea es una irrigación donde todo crece... Entonces la gente apuesta a eso. El clima es apto para todo, crianza de animales… en 40 días ya está el pollo. Entonces a eso se van, el clima es muy bueno acá… La producción crece rápido, al año sacas tres cosechas.*”

*“Firstly, this is an irrigation, an irrigation where everything grows. The people count on this. The climate is appropriate for everything, raising animals... in 40 days the chickens are ready. For this they come, the climate is very good... The production grows quickly, you have three crops a year.”*

*Changes in landscape, land ownership, and housing* - Participants described El Pedregal, at the initiation of the irrigation project, as a desert. There were no houses, and all the people used to live in the *parcelas.* The *parcela* owners (locally called “settlers” or ‘colonists’), hired the migrant farmers to live and work on their lands. The first populated area in El Pedregal was the La Colina neighborhood (one of the tree neighborhoods where triatomine infestation was detected), and the first constructions there were dedicated as “sedes” (headquarters) for a cooperative, the agrarian bank, and to a milk company, which used to buy milk from the farmers and, through their headquarters in La Colina, to make the respective payments. Over time, people applied for formal tenancy lots (land title or similar recognition). The time frame between land acquisition and tenancy is reported to be long in the newest settled areas, particularly due to the lack of infrastructure. In order to push for permanent tenancy, land acquisition was sometimes conditioned on its immediate occupation. As arriving migrants had few resources, they constructed shelters for themselves with fragile materials (*e.g.*, “*esteras*” which are woven straw mats), but as they gradually become settled, with titles of land ownership or access to electricity, they invested in materials for more permanent house construction, which generates job opportunities for those in construction. First local schools, health facilities, and food markets were also recalled being constructed with *esteras*, and rocks, replaced by plywood.

The absence of permanent neighbors is recalled in the past, and still reported in the present in newly settled areas (where triatomine infestation was also detected), where at the time of interviews, there is still no public water supply.

*“...compramos el lote y nos dijeron “tienen que vivir en el lote porque si no se lo quitamos”... Así, cuatro esteras eran todas las casas, tampoco no estaba cercado nada… Ya los que tienen platita cercábamos.”*

*“...we bought the lot and they told us ‘you have to live on the lot because if not we’ll take it’... So all the houses were just four esteras [Woven reed mats], there was nothing around the lot. Now people with a little money close their lot in.”*

*“Al comienzo compramos esteras… fue duro, yo dormía con una estera que doblaba, doblamos una estera y allí abajo dormían mis hijos… Ha sido difícil… como todos comenzamos así…”*

“*At first we bought esteras [Woven reed mats]... it was hard, I slept on a doubled-over estera, we folded an estera and my children slept below... It has been hard... like everyone we started like that...”*

*“...Ni siquiera el mercado era así no más, era puras telitas no más, la posta tambien ya que era de esteras, ni siquiera era así de concreto, rustico nomas era, después hicieron como de madera.”*

*“...Not even the market was like that, it was just some fabrics, the health post also was made of esteras, it wasn’t even concrete, just rustic, later they made it of wood.”*

*“...En el Módulo G era pampa no había ninguna casa, nosotros éramos una casa de esteritas, con un cuartito… ese colegio no existía, después poco a poco se formó, los salones eran de triplay y después ya lo construyeron. Igual la gente no vivía, no teníamos vecinos nosotros, ahora tampoco tenemos…”*

*“... Modulo G was empty land, not a single house. Ours was a house of little esteras, with a little room... the school wasn’t there, later bit by bit [the urbanization] formed, the rooms were of plywood and later they constructed them. Still people didn’t live here, we didn’t have any neighbors, we still don’t...”*

*Occurrence of dangerous insects and experiences regarding triatomine and bed bug infestation* - When asked about occurrences of dangerous insects in their community, participants recalled seeing triatomines at their homes among other insects and arachnids (*e.g.*, spiders, scorpions, flies, mosquitoes, ticks). The oldest inhabitants reported triatomine infestation in different neighborhoods of El Pedregal (*e.g.*, Pedregal Norte, Alto, Colina, Malvinas, and in the *parcelas*, particularly in the animal paddocks) and in the neighboring locality of Santa Rita de Siguas in the late 1980’s/early 1990’s. Frequent insecticide spraying campaigns are recalled having controlled triatomine infestations.

Migrants demonstrated knowledge regarding risk factors for triatomine infestation, particularly related to poor housing conditions and breeding animals near human dwellings. Participants knew they had to report infestation at the health center, and posters and flyers displaying pictures of triatomines were mentioned as sources of information about how to identify the insect and how to report infestation.

*“…Le voy a decir en qué tiempo, en 1986, 1987, había harto. Yo vivía en una parcela, trabajaba en una parcela, y de ahí cuando venía en la parcela misma había harto y acá en Pedregal se quejaban bastante de la Chirimacha. Hubo una campaña de fumigación, fumigaban seguido, seguido y entonces ha desaparecido.”*

*“...I’ll tell you when, in 1986, 1987, there were tons. I lived in a parcel, I worked in a parcel, and when I arrived on the parcel itself there were tons and here in Pedregal the people complained a lot about Chirimachas. There was a fumigation campaign, they fumigated frequently, after that the bugs disappeared.”*

*“…yo la conozco a la Chirimacha porque cuando vivíamos en el valle [Valle de Siguas], ahí sí hemos visto bastantes… En el valle hay campaña fuerte sobre el mal de Chagas, porque hay harta Chirimacha… La mayoría de casas eran de adobe y ahí era donde vivían las Chirimachas. El tema es que en el Valle teníamos todos nuestras casas pegadas al cerro, y pues los cerros son de esa tierra colorada y siempre había. Aunque en nuestra casa no había mucho porque como yo criaba gallos siempre fumigaba. Pero un año fueron donde la vecina que criaba cuy en el techo de su casa, de cemento se hizo ella su casa, supuestamente más higiénico que mi casita que era de adobe, y entonces fumigaron ahí en el conejero y waaa bajaban, parecía procesión del señor de los Milagros, en cantidad. Nunca vimos tantas Chirimachas como ese día que fumigaron donde la vecina…”*

“I knew of the Chirimacha because, when we lived in the valley, we used to see lots... In the valley [The Valley of Siguas] there are strong campaigns for Chagas disease, because there are tons of Chirimachas. The majority of the houses were of adobe and in the adobe lived the Chirimachas. The thing is, in the valley we had all our houses tied to the hillside, and well, the hills were of that red earth and there always were Chirimachas. Although in our house there weren’t many because, as I raised roosters, I always fumigated. But one year they went to the neighbor who raised guinea pigs on the roof of her house. She made her house out of cement, supposedly more hygienic than my house that was of adobe, and then they fumigated her house in her guinea pig pens and waaaa how they came down! It looked like the procession of the Señor de los Milagros*, tons. We never saw so many Chirimachas as that day they fumigated the house of our neighbor.”

*The procession of the Señor de los Milagros is a religious procession that occurs in Peru each October

*“…era una cosa negrita grandecita, pero era como su barriguita bien hinchadita, y mire yo no conocía antes. En la pared la vi, cuando prendimos la luz… qué será eso no conozco, qué animal será… como siempre íbamos con mi hijita a la posta, siempre íbamos ahí he visto un papelote, que decía Chirimacha y que produce una enfermedad, Chaga* [sic]*. Entonces ahí lo dejé.*”

*“It was a big black thing, with a kind of swollen belly, I looked at it, I had never seen it before. I saw it on the wall when we turned on the light... What could it be? I didn’t know, what animal could it be... As I often went with my daughter to the health post, I went there and I had seen a sign that said ‘Chirimacha’ and that it produced a disease, Chaga* [sic]*. So I left it there.”*

“*...Y le conocí acá porque como donde dormía era de esteras, cuando dormía me caminaban por encima, en la cara, y prendía la luz y veía a la Chirimacha...*”

*“...I came to know it here because where I slept was made of esteras, while I slept, they walked all over me, on my face, and I’d turn on the light and see the Chirimachas...”*

Participants reported having heard about bed bug infestations from their neighbors, friends, and health workers in door-to door prevention campaigns. Health promotion materials (posters) were reported as source of information to both identify *Chirimachas* and report infestations by internet.^33^ Inhabitants who had their houses infested by bed bugs, reported use of different products and practices to control infestation, such as: use of “Sapolio^®^ aerosol”, “Bolfo^®^” (Propoxur- carbamates), chlorine, and cleaning activities (taking out the mattress to expose it to the sun). Some of those who experienced infestation believe that bed bugs may transmit diseases but did not know which one.

*“...me mudé recién hace un año y aparecía ronchas… y yo lava, limpia y no había nada, no se ve “¿Dios qué es esto?” y una amiga me dijo “el chinche te pica así y en un solo sitio, harto en un solo sitio”. Me pidió que le enseñara la roncha y me dijo que era Chinche, yo pues asustada, le pregunté ¿Qué tengo que hacer? Entonces había un letrero en el kiosquito que decía “Llamar cuando tienes Chirimachas” Le he llamado pues y le he dicho “Por favor no sé lo que tengo” entonces han venido y me han revisado todito así, la señorita desarmó la cama y ahí dentro estaban. Tenía que echarle lejía, echarle agua hervida, ya no quería dormir ahí.”*

*“...I moved recently, a year ago, and bumps appeared... I washed, cleaned and there was nothing, you can’t see them “G-d what is this? and a friend told me ‘the bed bugs bite you that way in one spot, lots in one spot’. She asked me to show her the bumps and she told me that it was bed bugs, I was frightened, I asked her ‘What do I have to do? Then she told me about a sign on a kiosk that said, ‘Call when you have Chirimahcas’. I called then and I told them ‘Please, I don’t know what I have’ so they came and inspected everything, the women took apart my bed and there they were, inside. I had to use bleach, boiling water; I didn’t want to sleep there anymore”.*

“*...Porque todo animalito pasa de vecino a vecino…Si hubiera acá esos bichitos (Cimex) uso el Bolfo* ^*®*^ *, no hace daño pues, y sacar pues al sol, más que todo sacar al sol hasta para las pulgas, antes había harta pulga, teníamos que sacar las frazadas al sol y como el sol calienta la frazada las pulgas salen a caminar, salen a buscar un sitio donde ocultarse… porque en la noche no se ve, pica pero no se ve… En cambio, sobre la frazada, lo sacas al sol, lo extiendes y ahí si sale. Pueden venir de la sierra en las cosas que los viajeros traen, puede venir en la ropa, en la frazada, en cualquier cosa que ellos traigan pueden venir y yo tengo entendido que ese animalito se adecua, se adaptara al ambiente, del frío al calor…*”

*“...Because every little animal passes from neighbor to neighbor... If they had here those bugs I’d use Bolfo* ^*®*^ *, It doesn’t cause harm, and put everything out in the sun, more than anything, put things in the sun, like for fleas, there used to be tons of fleas, we had to put the blankets out in the son, and as the sun warmed the covers the fleas walked off, they left to search for a place to hide themselves... because at night you can’t see them... But if you spread the covers out in the sun they leave. They might come from the mountains in the things that travellers bring, they could come in clothing, in the bedding, in anything that they bring they could come and I understand that this little animal adapts itself to any environment, from cold to hot...”*

Although participants had their houses IRS treated, 79.3% (23/29) reported using other insecticide products against triatomines, bed bugs and other synanthropic insects, such as: Butox^®^ (deltamethrin); Raid^®^; Sapolio^®^; Baygon^®^. Two respondents reported using the agricultural pesticide Lannate^®^ (Methomyl-carbamate),^34^ applied indoors and outdoors (against triatomine infestations), and applied directly on a dog (for fleas).

*“...si es cantidad* [de triatominos] *tenemos que fumigar obligatoriamente… Esas que utilizamos en las parcelas, esas bombitas que botan presión, nebulizado con eso, dependiendo un producto especial para la Chirimacha no me acuerdo. Hay varios productos que tienen varios nombres, nosotros utilizabamos el Lannate* ^34^ ^)^ *que era un veneno fuerte, con eso fumigábamos y teníamos que estar fuera de la casa por lo menos 12 horas.”*

*“…if there were a lot of [triatomines] we definitely have to fumigate... What we used was what we used on the fields, those sprayers that work by pressure, spraying with those, relying on a special product for Chirimachas, I dont remember which. There are various products that have various names, we used ‘Lannate* ^34^ ^)^ *[Methomyl], which was a strong poison, we sprayed with that and we had to be outside the house for at least 12 h.”*

*“En La Joya si fumigábamos, pero no me acuerdo el nombre. Acá no hemos fumigado… Yo tiempo atrás tuve un perro… Entonces lo agarró las pulgas y no se dejaba curar… le he echado de todo hasta que una vecina me aconsejó usar Lannate, que se utiliza para la agricultura, y recién pude matar las pulgas.”*

*“In La Joya we sprayed, but I can’t remember the name [of the insecticide]. We haven’t sprayed here... A long time ago I had a dog... It got fleas and couldn’t get rid of them... I tried everything until a neighbor told me to use Lannate, which they use on the farms, and then I could kill the fleas.”*

*Assessment of household development via satellite images* - Houses infested by *T. infestans* were constructed significantly earlier than control houses (Wilcoxon rank-sum test: W = 33, p = 0.02). No statistical difference was observed between median years of construction when we compared houses infested by *Cimex* sp. and control houses (W = 6.5, p = 0.07) [Supplementary data (Table)].

**Fig. 2:**
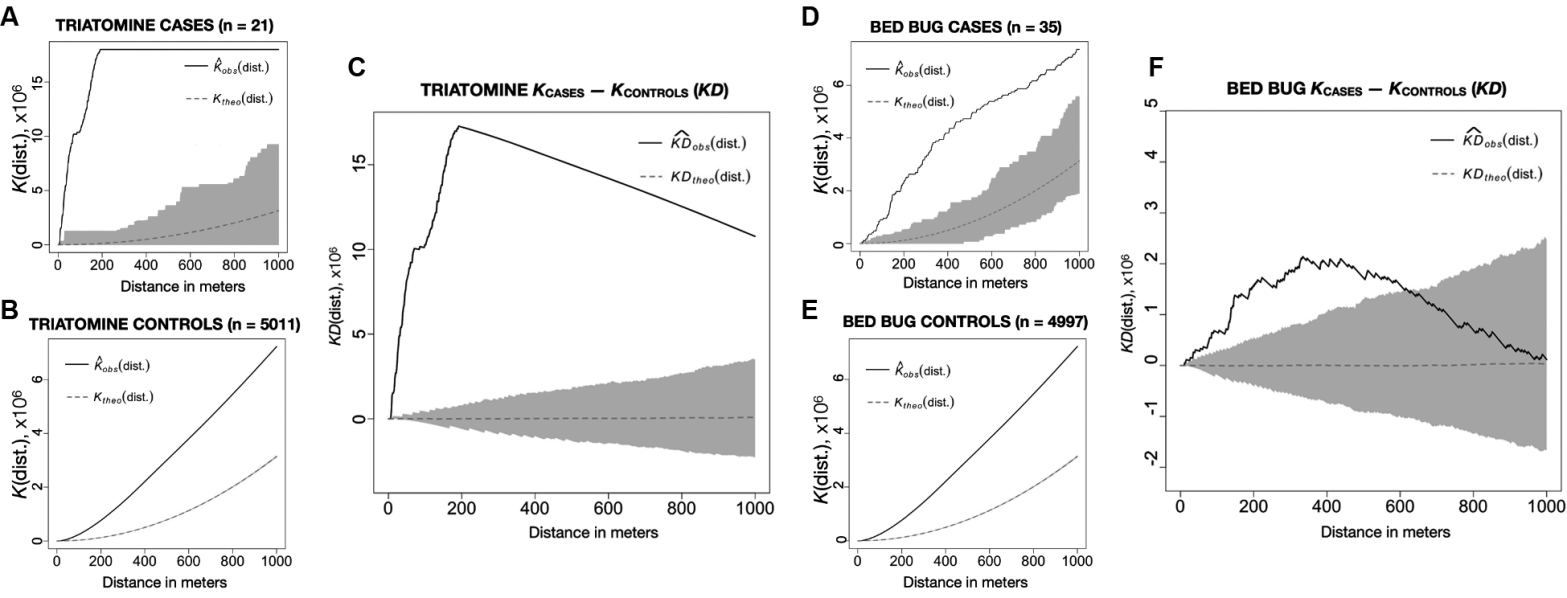
Ripley’s K functions measuring spatial clustering for triatomine cases (a) and controls (b) and bed bug cases (d) and controls (e), as well as the difference in K functions (KD) for cases and controls for triatomines (c) and bed bugs (f) for a distance of 0-1000 m. Black lines indicate observed values, dotted red lines represent null hypothesis of no clustering, and grey shading represent 95% tolerance envelopes. Significant clustering for triatomine infestations is detected at all distances, while clustering for bed bug infestations is detected for distances less than ~ 600 m.

*Spatial analysis* - The difference in KD indicated significant clustering of both triatomine and bed bug infestations at various spatial scales (Fig. 2). The Ripley’s *K* functions of observed case and control data for both infestation types showed significant clustering across all distances considered (Fig. 2A, B, D, E), suggesting that the locations of inspected houses exhibit significant clustering at baseline. However, the observed *KD* functions were positive up to 1000 m for triatomines and up to 600 m for bed bugs, which suggests that triatomine and bed bug infestation exhibit clustering over and above any clustering observed in non-infested homes (Fig. 2C, F).

## DISCUSSION

Throughout history, human populations have employed migration as a survival strategy in response to various challenges, including the impacts of climatic events,^35^ ^,^ ^36^ ^,^ ^37^ food insecurity,^38^ ^,^ ^39^ or pursuing improved living conditions.^40^ Governmental policies focused on the implementation of extensive infrastructure projects, extractive industries, and agribusiness activities, can generate a need for additional labor force,^41^ often leading to migration into newly urbanizing areas. The resulting, often haphazard, settlement promotes conditions conducive to disease transmission by connecting hosts, pathogens, and vectors.^42^

The *Chirimacha*, *T. infestans*, successfully invaded urban and peri-urban areas in Arequipa, Peru;^10^ ^,^ ^43^ Cochabamba, Bolivia;^44^ and Argentina;^45^ ^,^ ^46^ complicating regional efforts to eliminate vector-borne Chagas disease.^43^ ^,^ ^46^ Here, we describe the migration histories that contributed to population growth in El Pedregal, and we assess residents’ perceptions regarding urban development, and their experiences regarding triatomine and bed bug infestations.

The Majes irrigation project was designed to supply water resources for the establishment of agribusiness companies in El Pedregal.^20^ These companies had a huge impact on local farming practices and economy, by introducing new products and agriculture practices, fixing prices for milk, and eventually leading to a shift in the main products in this region: from cattle and dairy to fruits and vegetables. Their establishment required a permanent labor force, which attracted subsistence farmers from across the country. These populations declared different reasons for migrating to El Pedregal; while some of them were seeking opportunities to own a house or to improve their living conditions, others were only searching for a new place to keep working the land. Subsistence farmers assumed the agribusiness companies would secure a permanent work offer, a different reality than they faced elsewhere, where their farming practices were vulnerable to seasonality, local trade dynamics, and to adverse climate events. Coming to a desert, these migrants arrived with few resources; only enough to live in shelters constructed of woven reeds and other poor-quality materials. Over time, working in the fields with consistent income, they constructed more permanent houses. Most of the houses, however, continued to incorporate precarious materials, such as unmortared stones or “*esteras*” (woven reeds), providing numerous hiding places for triatomines. The prolonged precarity of houses in the area provided stable environments for introduced triatomines to establish colonies.

Through interviews and satellite images, we assessed house construction and the expansion of the settlement over space and time. We observed that not all constructed houses were immediately inhabited, as evidenced by the nearly one-to-one inhabitant-to-dwelling ratio in the 2017 Peruvian census for Majes district, which reported 60,108 inhabitants and 58,298 dwellings registered.^47^ This low ratio is likely due to the large number of dwellings that have been ‘staked out’ but not fully constructed. In our door-to-door surveys in the fairly established areas, we found that approximately 15% of houses were uninhabited. The long interval between the tenancy of space and its effective use was also mentioned by participants in the in-depth interviews. Uninhabited houses might act as a limiting factor for triatomine dispersion, as no sources of food are available in these structures nor in the desert around them.^48^ The uneven construction may thereby have restricted triatomines to a small number of households, spatially clustered, in the older sections of El Pedregal.

Households infested with *T. infestans* tended to be older, in areas where migrants had come many years ago. In contrast, infestation by bed bugs presented a different pattern, more dispersed in comparison to those infested by *T. infestans*. Bed bug infestation was not associated with the age of the home, nor the year of first construction of the structure. Although bed bug infested households were more spatially clustered than would occur by chance, they were much more widely dispersed across El Pedregal than the triatomines. Bed bugs are likely less dependent on active dispersal in El Pedregal, and, by being carried passively in belongings, less affected by the barriers of uninhabited houses, barren vacant lots, and streets.

Awareness of bed bug infestation was amplified through communication amongst community members and health workers, as well as the new online reporting system ‘AlertaChirimacha’.^33^ Still, knowledge regarding infestation control is low, and indiscriminate use of insecticides products were reported against domestic infestations of bed bugs, triatomines, and other synanthropic insects. Similar results were observed in peri-urban neighborhoods of the metropolitan Arequipa.^49^

Although householder application of insecticides was associated with lower risk of infestation by *T. infestans* in endemic areas in Argentina,^45^ insecticide misuse might decrease susceptibility of vector populations to these and related products.^50^ Aerosol insecticide formulations, in particular, are easily available for purchase in Peru, and their use is widespread. These products often combine different insecticides and synergists agents and are unlikely to control triatomine infestations effectively. Use of aerosol products has shown to increase resistance to pyrethroids in populations of *Aedes aegypti* in Mexico.^50^ Health educational campaigns to improve community knowledge regarding methods to control synanthropic insects, particularly for bed bug infestations, could help to prevent misuse of insecticide products. However, until a safe and affordable alternative is made available for bed bugs, as it is for triatomines, it is hard to imagine the bed bug situation improving.

We speculate, based on our synthesis of spatial entomological data and our interviews, that the *Chirimacha* populations we detected and eliminated arrived at El Pedregal rather recently, and were hindered from dispersing by the numerous uninhabited lots in the growing city. It is possible that the insects are remnants of much earlier infestation processes that our participants recounted, but we believe that alternative explanation is less likely, because it is hard to imagine the insects being constrained to such small foci of infestations for such a long time. Indeed, in the neighboring district of La Joya, *Chirimachas* redispersed following control in the 1990s to infest over a quarter of households.^51^ It is also possible that the actions of residents applying their own insecticides prevented the further dissemination of *Chirimachas*, though again it is hard to imagine those activities would be effective and uniform enough for long term control. If we are correct our results are concerning - they suggest that as El Pedregal develops further, and the empty lots are filled in, the new city will be highly susceptible to triatomine infestations.

The historic and current triatomine infestations, which occurred about 30 years apart, seem to differ in their dispersal and density and the settings of space occupation. The former is recalled in interviews as a widespread event, affecting different neighborhoods, houses, and animal corrals at high densities. Housing conditions in those days were described as improvised constructions, using poor quality materials. The infestations we observed in Pedregal, however, were extremely spatially clustered, of low insect densities and only in a few houses that were constructed many years ago. Still, those houses were infested at low densities, and infestation spread was limited, likely by the environmental barriers created by unoccupied, or very sparsely occupied, lots, interspersed with more permanent and, for the insects, habitable, dwellings. Housing conditions, particularly construction materials and crowding, which provide availability of refuges and source of food for triatomines, are limiting factors for domestic infestation by triatomines, density of its colonies, and infection by *T. cruzi* across many contexts.^9^ ^,^ ^10^ ^,^ ^52^ ^,^ ^53^ ^,^ ^54^ ^,^ ^55^ In the interval between the two (re-)introduction events, changes in housing materials and conditions in El Pedregal may not have made houses entirely refractory to infestation, but they hampered infestation, dispersal, and density of the insects. None of the triatomine specimens were infected by *T. cruzi*. The lack of *T. cruzi* in these insects is in line with our observations in the city of Arequipa, where we have not detected parasite-infected insects in areas with very sparse vector infestation.^43^ ^,^ ^56^

This study had several important limitations including the usual issues with imperfect detection of insects through household search,^54^ ^,^ ^57^ and the various biases that can interfere with qualitative methods. Most of the fieldwork reported in this study was conducted during significant severe acute respiratory syndrome (SARS)-Covid 19 transmission. The discrepancy between the 12,129 households interviewed (at the door) and the 5,164 that permitted a full entomological inspection was due to concerns among participants with having personnel enter their homes.

Large government decisions involving water and changes in land use can have outsized impacts on the lives of farmers and other laborers, and these impacts can reverberate through society, leading to mass migration and, ultimately, increasing populations’ vulnerability to disease.^58^ ^,^ ^59^ ^,^ ^60^ ^,^ ^61^ The cost of these decisions is ultimately borne by those with the least recourse to bear them.^62^ ^,^ ^63^ Migration has been also driven by adverse climate conditions, such as flooding, as reported by a migrant from Puno. This phenomenon is expected to increase in the following years. As the climatic crisis changes the global water cycle,^64^ responsible and participatory management to ensure water access for all and protection of natural ecosystems, is critical.

Ironically for a city that grew around an irrigation project, many residents of El Pedregal lack access to clean water.^20^ Without running water many resort to storing water in barrels, which creates risk for the emergence of *Ae. aegypti* and Dengue virus,^65^ currently expanding throughout southern Peru.^66^ The complex forces of migration and poverty make El Pedregal especially vulnerable to vector-borne diseases. A hypervigilance among communities and health authorities is needed to rapidly respond to pathogens as they emerge.
