# Supplementary material for: Irrigation, migration and infestation: a case study of Chagas disease vectors and bed bugs in El Pedregal, Peru
Source: Mem Inst Oswaldo Cruz. 2024 Sep 2;119:e240002. doi: 10.1590/0074-02760240002 (PMC11370655; doi:10.1590/0074-02760240002)
Supplement: Supplementary file 1 [file 1678-8060-mioc-119-e240002-s.pdf]

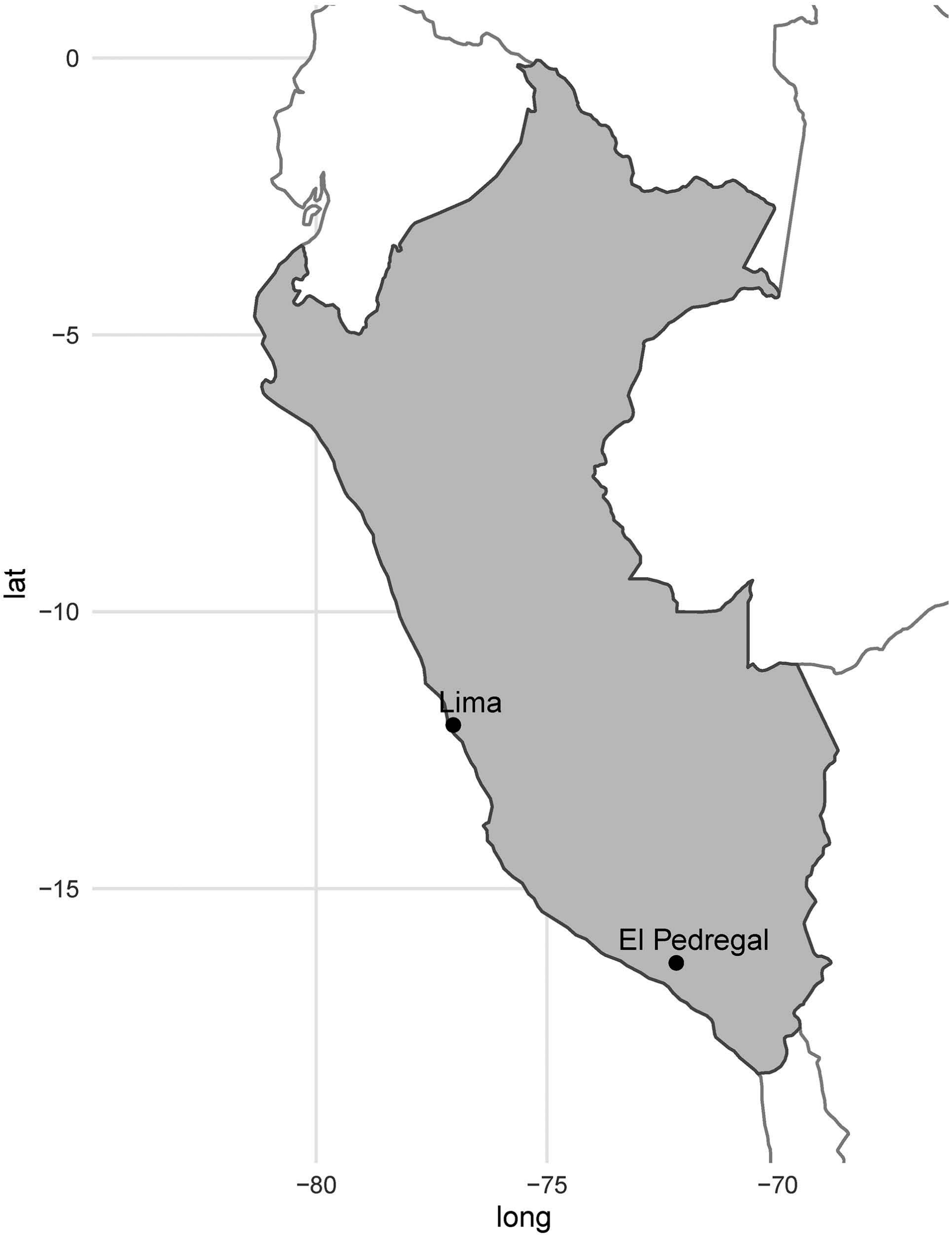

Map of El Pedregal, Peru.

TABLE

Number of houses by year of first construction, as observed through remote sensing, according to infestation status

House groups	2004	2010	2012	2017	2018	2020	Total
Control	22	74	12	15	8	11	142
Triatoma infestans	14	5	1	1	0	0	21
Cimex sp.	15	14	3	1	0	0	33
Total	51	93	16	17	8	11	196

CC BY
